# HIV-1 Envelope Protein gp120 Promotes Proliferation and the Activation of Glycolysis in Glioma Cell

**DOI:** 10.3390/cancers10090301

**Published:** 2018-09-01

**Authors:** Gabriel Valentín-Guillama, Sheila López, Yuriy V. Kucheryavykh, Nataliya E. Chorna, Jose Pérez, Jescelica Ortiz-Rivera, Michael Inyushin, Vladimir Makarov, Aníbal Valentín-Acevedo, Alfredo Quinones-Hinojosa, Nawal Boukli, Lilia Y. Kucheryavykh

**Affiliations:** 1 Department of Biochemistry, Universidad Central del Caribe, School of Medicine, Ave. Laurel, Santa Juanita, Bayamon, PR 00956, USA; gabriel.valentin2@upr.edu (G.V.-G.); yuriy.kucheryavykh@uccaribe.edu (Y.V.K.); 115jperez@uccaribe.edu (J.P.); jescelica.ortiz@upr.edu (J.O.-R.); 2 Biomedical Proteomics Facility, Department of Microbiology and Immunology, Universidad Central del Caribe, School of Medicine, Ave. Laurel, Santa Juanita, Bayamon, PR 00956, USA; sheila_natalie@yahoo.com (S.L.); nawal.boukli@uccaribe.edu (N.B.); 3 Department of Biochemistry, University of Puerto Rico, School of Medicine, San Juan, PR 00936, USA; nataliya.chorna@upr.edu; 4 Department of Physiology, Universidad Central del Caribe, School of Medicine, Ave. Laurel, Santa Juanita, Bayamon, PR 00956, USA; mikhail.inyushin@uccaribe.edu; 5 Department of Physics, University of Puerto Rico, Río Piedras Campus, San Juan, PR 00931, USA; vmvimakarov@gmail.com; 6 Department of Microbiology and Immunology, Universidad Central del Caribe, School of Medicine, Ave. Laurel, Santa Juanita, Bayamon, PR 00956, USA; anibal.valentin@uccaribe.edu; 7 Department of Neurologic Surgery, Mayo Clinic, 4500 San Pablo Road South, Jacksonville, FL 32224, USA; quinones-hinojosa.alfredo@mayo.edu

**Keywords:** glioma, HIV, gp120, glycolysis

## Abstract

Patients infected with human immunodeficiency virus (HIV) are more prone to developing cancers, including glioblastomas (GBMs). The median survival for HIV positive GBM patients is significantly shorter than for those who are uninfected, despite the fact that they receive the same treatments. The nature of the GBM–HIV association remains poorly understood. In this study, we analyzed the effect of the HIV envelope glycoprotein gp120 on GBM cell proliferation. Specifically, we performed cell cycle, western blot, protein synthesis and metabolomics analysis as well as ATP production and oxygen consumption assays to evaluate proliferation and metabolic pathways in primary human glioma cell line, U87, A172 cells and in the HIVgp120tg/GL261 mouse model. Glioma cells treated with gp120 (100 ng/mL for 7–10 days) showed higher proliferation rates and upregulation in the expression of enolase 2, hexokinase and glyceraldehyde-3-phosphate dehydrogenase when compared to untreated cells. Furthermore, we detected an increase in the activity of pyruvate kinase and a higher glycolytic index in gp120 treated cells. Gp120 treated GBM cells also showed heightened lipid and protein synthesis. Overall, we demonstrate that in glioma cells, the HIV envelope glycoprotein promotes proliferation and activation of glycolysis resulting in increased protein and lipid synthesis.

## 1. Introduction

Patients infected with human immunodeficiency virus (HIV) are more predisposed to developing cancer, including glioblastoma (GBM) [1,2] and ten percent of patients with acquired immune deficiency syndrome (AIDS) have brain tumors. Although the majority of these tumors are central nervous system lymphomas, glioma tumors arise as well [3]. Multiple medical reports in HIV/AIDS patients indicate that GBM occurs at a higher frequency (5.4- to 45-fold increase) [4,5,6,7] and at a younger age [8] in individuals at various stages of HIV infection than in the general population. On average, GBM tumors appear approximately 3 years after initial HIV infection [2]. Additionally, the median survival rate in GBM-HIV-infected patients is shorter (an average of 8 months) than in GBM-non-infected patients (an average of 14 months) despite receiving the same treatment [2,3,9,10]. CD4+ cell count at initial diagnosis of GBM is not correlated with survival suggesting that increased aggressive tumor behavior is not a direct outcome of immune deficiency [8].

The nature of the GBM–HIV relationship is not well understood. The stimulatory effect of HIV infection on glioma tumor development has been associated with reduced immune surveillance [11,12]. However, immune incompetence has not been clearly shown to underlay glioma tumor development and progression [7,13,14]. While the incidence of some malignancies among HIV-infected individuals has declined with Highly Active Antiretroviral Therapy (HAART), it remains elevated compared with uninfected population suggesting that overall immune deficiency is not the only cause [15,16,17]. Moreover, HIV is not found in glioma tissues of patients diagnosed with GBM [2]. It has been shown that some human GBM cell lines have the ability to suppress HIV infection by secreting molecules that inhibit HIV attachment to target cells, while other GBM cell lines do not [18]. The predominant HIV target cells in the brain are microglia and macrophages, while other cells such as astrocytes, oligodendrocytes, neurons and microvascular cells tend to be mostly resistant [19,20,21].

It has been shown that HIV infection promotes the development of glial tumors through a set of factors that includes the activation of oncogenes, the impairment of immune defenses and the production of growth factors and cytokines capable of inducing astrocytosis [21,22,23,24]. Additionally, tumor cells can be directly exposed to HIV proteins such as gp120, which can be secreted by infiltrated and infected microglia and astrocytes [25,26]. 

HIV is believed to enter and infect the central nervous system through the interaction between the envelope protein gp120 and the CCR5 or CXCR4 receptors expressed on macrophages but some neurons and astrocytes are thought to express these receptors as well, which can result in their subsequent infection [3,24]. GBM cells can also express CXCR4 and CCR5 and activation of these receptors is known to promote cell survival and cell cycle progression [27,28,29,30,31]. We hypothesize that despite some innate resistance to HIV infection, glioma cells can interact with the HIV envelope protein gp120 and this interaction promotes cell proliferation and tumor growth.

In this study, we investigated the effect of the HIV envelope protein gp120 on glioma cell growth and survival. We showed that continuous treatment of the U87 and A172 glioma cells and the primary human glioma cell line 965 with gp120 for a period of 10 days resulted in increased proliferation, migration and survival. Through a combination of western blot and metabolomics analysis we also detected the activation of glycolysis as well as protein and fatty acid synthesis metabolic pathways in these cells. Finally, using a HIVgp120tg/Gl261 mouse glioma implantation model, we demonstrated that animals expressing gp120 in their brain develop bigger tumors and have shorter median survival than their wild type littermates (WT).

## 2. Results

### 2.1. Gp120 Stimulates Proliferation and Migration in Glioma Cells

**Figure 1:**
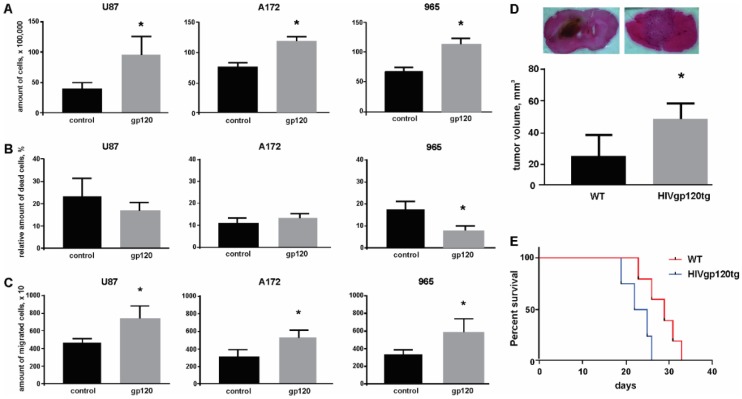
gp120 stimulates viability and migration and reduces cell death in glioma cells. Experiments were performed for untreated glioma cells and cells continuously treated with gp120 for 10 days. U87 and A172 cell lines and 965 primary glioma cells were investigated. Live and dead cells were counted with the use of trypan blue staining. (**A**) Cell viability was evaluated as the total number of live cells. (**B**) The proportion of dead cells was evaluated as the percentage of the total number of cells. (**C**) Migration assays were performed using Transwell membranes for 5 h of migration toward 5% serum-containing medium in the lower compartment. The total number of cells that had migrated to the lower compartment was determined and is represented in the graph. (**D**) H&E staining of brain sections encompassing glioma tumor and quantification of tumor size in wild type (WT) and HIVgp120tg mice. (**E**) Survival analysis, performed for WT and HIVgp120tg mice following GL261 glioma cells implantation. Mean ± S.E. and significant differences from control (*) are shown (*p* < 0.05). Unpaired *t*-tests were used to determine the significance between groups. Comparison of survival curves was performed by use of long-rank Mantel-Cox test. Six repeated experiments (*n* = 6) were used for statistical analysis.

Our initial observations on the effect of gp120 in the proliferation of glioma cells came from a trypan blue exclusion assay. After treating cells with gp120 at 100 ng/mL for 10 continuous days we observed an increase in the number of viable cells in all cell lines investigated, including the 965 primary glioma cell line (Figure 1A). The gp120 concentration we used has been reported in the literature as effective for inducing signaling in glioma cells and it is also consistent with the gp120 serum concentration in HIV patients [32,33]. A significant difference in cell growth between gp120-treated and untreated cells was not observed at earlier time points (less than 5 days of treatment) (Supplemental Figure S1A). In addition, 965 cells showed a reduction in the amount of cell death (Figure 1B). This effect on cell survival was not observed in other cancer cell lines tested, including Jurkat T cells, in where gp120 showed instead a toxic effect (Supplemental Figure S2).

Treating glioma cells with gp120 also had a positive effect in migration. In a transwell migration assay, gp120-treated glioma cells showed a greater migration propensity than untreated cells (Figure 1C).

In vivo studies using the HIVgp120tg mice, which expresses the HIV gp120 glycoprotein in the central nervous system (CNS), demonstrated that upon implantation of GL261 mouse glioma cells animals develop bigger brain tumors compared to their WT littermates (Figure 1D). Additionally, HIVgp120tg mice had 15% shorter survival rates (23.5 days) when compared to WT animals (27.5 days) (Figure 1E). This HIVgp120tg mouse model has been previously described and characterized [34,35,36]. Expression of gp120 in brain and implanted tumor in HIVgp120tg mice is shown in Supplemental Figure S3.

**Figure 2:**
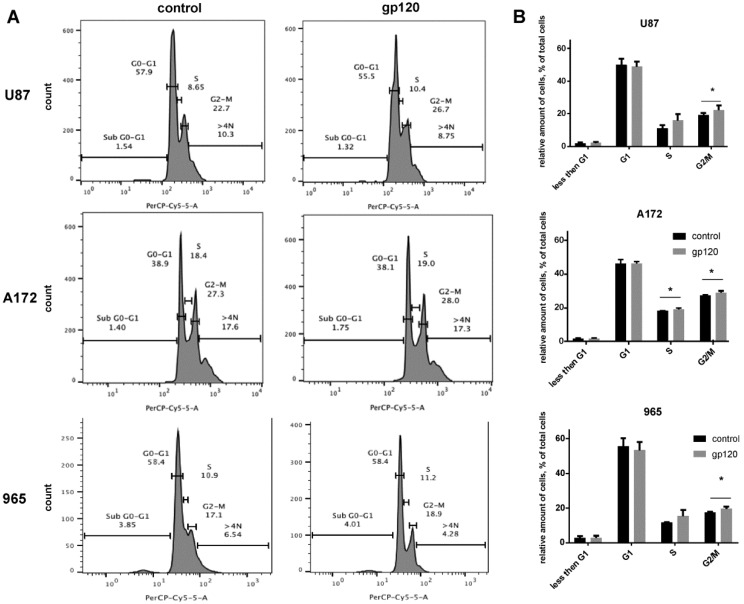
Gp120 stimulates proliferation of glioma cells. Cell cycle analysis was performed by analyzing cells stained with 7-aminoactinomycin D (7AAD) with flow cytometry. The percentage of cells in the G0/G1, S and G2/M phases was determined based on DNA content. Experiments were performed for untreated glioma cells and cells continuously treated with gp120 for 10 days. U87 and A172 cell lines and 965 primary glioma cells were investigated. (**A**) Histograms and (**B**) bar graphs represent the total distribution of cells at different phases of the cell cycle. The proportion of cells at each phase of mitosis is shown as a percentage of the total number of cells. Mean ± S.E. and significant differences from control (*) are shown (*p* < 0.05). Unpaired *t*-tests were used to determine the significance between groups. Four repeated experiments (*n* = 4) were used for statistical analysis.

Cell cycle analysis using flow cytometry confirmed and further extended our results on cell proliferation showing that glioma cells treated with gp120 have a higher frequency of mitosis than untreated cells (Figure 2). Despite the different basal proliferation rates in the glioma cell lines investigated (the average percentage of cells at the G2/M phase of mitosis was 19 ± 0.64% of the total number of cells for U87, 27 ± 0.25% for A172 and 17 ± 1.76% for 965 cells), a 7–10-day treatment with gp120 resulted in an increase in the percentage of cells at the G2/M phase to 20.6 ± 0.51%, 28.5 ± 0.32 and 18.8 ± 1.6, respectively (*n* = 4). Consequently, the average increase in the percentage of cells at the G2/M phase in gp120-treated cells over untreated cells was 1.6%. For cells in the S phase we only observed a significant increase in A172 cells (18.2 ± 0.18% in untreated vs. 19.1 ± 0.7% gp120-treated). U87 and 965 showed insignificant increase in this population in response to gp120 treatment (11.02 ± 2 in untreated vs. 15.8 ± 3.9 in gp120-treated U87 cells and 11.73 ± 0.2% in untreated vs. 15.4 ± 3.6% in gp120-treated 956 cells). For all cell lines investigated, we observed no difference in response to gp120 in the number of cells in the sub-G1 phase, which is indicative of cell undergoing apoptosis. Taken together, our results demonstrate that the HIV-gp120 glycoprotein induces proliferation in glioma cells.

Based on these results we calculated the duplication time for cells treated with gp120 (*τ*_1_) and untreated (*τ*_2_).

During time *t*_0_, the number of duplication events can be determined as follows:

(1) $$N_{i}=\frac{t_{0}}{\tau_{i}};\;i=1,\;2\;(1—\text{gp120-treated},\;2—\text{untreated cells})$$

Taking *n* as the initial number of cells and *q*_1_/*q*_2_ as the ratio of cells at the G2/M phase with and without treatment with gp120, the final number of gp120-treated (*n*_1_) and untreated (*n*_2_) cells can be determined as follows:

(2) $$\begin{array}{l} n_{i}=n+q_{i}n+q_{i}\left(n+q_{i}n\right)+q_{i}\left(n+q_{i}n+q_{i}\left(n+q_{i}n\right)\right)\\ +q_{i}\left[n+q_{i}n+q_{i}\left(n+q_{i}n\right)+q_{i}\left(n+q_{i}n+q_{i}\left(n+q_{i}n\right)\right)\right]\\ +..\approx A_{i}e^{\alpha_{i}N_{i}}\\ i=1,\;2\\ q_{1}=0.206\;q_{2}=0.190 \end{array}$$

The values of *q*_1_ and *q*_2_ were obtained through direct summation of cell populations *n_i_* created in each growth step, presented by the simplest kinetics model described earlier [37], where *α_i_* is the parameter of kinetic model and Ni >> 1. Since we initiated the experiment with the same number of cells for both treated and untreated groups, A1 = A2. Thus, given that after 10 days the number of treated cells was twice the amount on the untreated group (Figure 1).

(3) $$\begin{array}{l} \frac{n_{1}\;}{n_{2}}=e^{\alpha_{1}N_{1}-\alpha_{2}N_{2}}=e^{\alpha_{1}\frac{t_{0}}{\tau_{1}}-\alpha_{2}\frac{t_{0}}{\tau_{2}}}=e^{\left(\frac{1}{{\tau^{\prime }}_{1}}-\frac{1}{{\tau^{\prime }}_{2}}\right)t_{0}}=2\\ \frac{1}{\tau \prime_{1}}=\frac{\alpha_{1}}{\tau_{1}}\\ \frac{1}{{\tau^{\prime }}_{2}}=\frac{\alpha_{2}}{\tau_{2}} \end{array}$$

The goal of this analysis is to find the ratio *τ*_2_/*τ*_1_. Taking into account dependence of *n_i_* obtained by direct summations of the respective *n_i_* rows, where values of *q_i_* are defined in (2) and further fitting both cases with *q*_1_ = 0.206 and *q*_2_ = 0.190 by exponential function (2), we found that *α*_1_ ≈ 0.190 and *α*_2_ ≈ 0.198.

**Figure 3:**
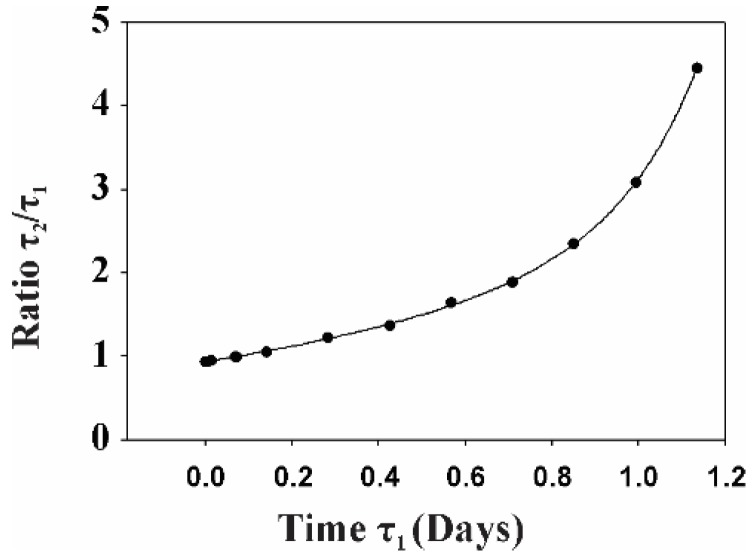
The dependence of *τ*_2_/*τ*_1_ on *τ*_1_, where *t*_0_ is constant and equal to 10 days.

Since Equation (3) consists of two unknown parameters, we carried out numerical analysis of the *τ*_2_/*τ*_1_ dependence on *τ*_1_, where *t*_0_ is a constant with a value set at 10 days. Taking into account Equation (3) we carried out numerical analysis, the results of which are shown in Figure 3.

As can be seen from this figure, if *τ*_2_/*τ*_1_ decreases, the ratio asymptotically approaches the 0.94 limit, while if *τ*_1_ increases, the ratio increases nonlinearly. Since the value of *τ*_1_ is unknown, we can make only a qualitative conclusion that the duplication time of untreated cells is larger than the time for gp120-treated cells for *τ*_1_ > 0.19 days. If *τ*_1_ = 0.19 days, a ratio of treated versus untreated cells of 2 is obtained at approximately 78 duplication cycles. For a culture containing an average of 20% of the cells in the G2/M phase, this results in 78 × 0.20 = 16 duplication events for each cell in the culture. Taking into account that, on average, 10% of the cells in the culture undergo apoptosis (Figure 1B), this number of duplication events corresponds to the average number of duplication cycles for glioma cells cultured for 10 days and confirms that a 1.6% difference in the percentage of cells in the G2/M phase between the groups provides a two-fold differences in the total number of cells at 10 days.

### 2.2. Gp120 Causes Upregulation in the Expression of Glycolytic Enzymes and Increases the Ratio of Glycolytic Index in Glioma Cells

As we recently published, quantitative Tandem Mass Tag (TMT) isobaric labeling quantitative proteomics analysis revealed that gp120 causes upregulation of a number of proteins involved in glycolysis and in the tricarboxylic acid (TCA) cycle in glioma cells including enolase 2 (ENO2), glyceraldehyde-3-phosphate dehydrogenase (GAPDH) and malate dehydrogenase [32].

**Figure 4:**
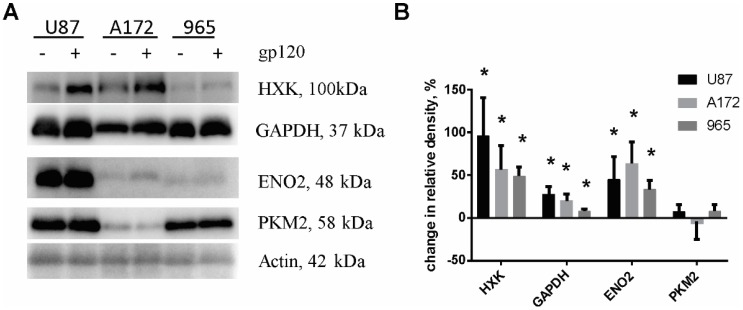
Gp120 upregulates expression of glycolytic enzymes in glioma cells. Western blot (**A**) and quantification of relative changes of glycolytic enzyme protein levels (**B**) for untreated and gp120-treated glioma cells. The bar graph shows the percent change in density of protein in gp120-treated relative to untreated cells. U87 and A172 cell lines and 965 primary glioma cells were investigated. β-actin was used as a loading control. The intensity of the chemiluminescent signal was corrected for minor changes in protein content after densitometry analysis of the India ink-stained membrane. Results are presented as mean ± S.D. with significant differences from control (*) (*p* < 0.05). An unpaired *t*-test was used to determine the significance between gp120-treated and untreated groups. Five independent experiments (*n* = 5) for each cell line were used for statistical analysis.

**Figure 5:**
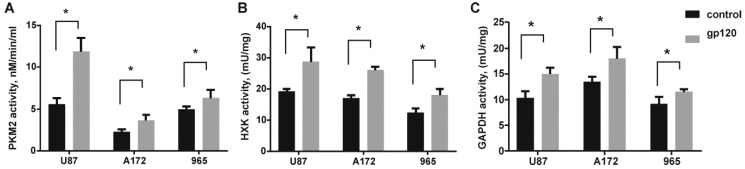
Gp120 increases the activity of glycolytic enzymes in glioma cells. Colorimetric/fluorometric pyruvate kinase (**A**), hexokinase (**B**) and glyceraldehyde 3-phosphate dehydrogenase (**C**) activity assays were performed in untreated U87, A172 and 965 glioma cells and the same cells continuously treated with gp120 for 7–10 days. Kinase activity was calculated as the amount of product (pyruvate, NADPH and NADH respectively) produced in a sample in 10 min. Mean ± S.E. and significant differences between control and gp120 treatment (*) are shown (*p* < 0.05). Unpaired *t*-tests were used to determine the significance between groups. Five independent experiments (*n* = 5) were used for statistical analysis.

In order to characterize the mechanisms underlying the increased proliferation of glioma cells treated with gp120, we validated expression levels of glycolytic enzymes by immunoblotting. A significant increase in protein expression was observed for hexokinase (HXK), ENO2 and GAPDH in all the glioma cell lines tested (Figure 4). At the same time, we observed increased activity of HXK and GAPDH (Figure 5). No elevation of pyruvate kinase M (PKM2) expression was observed. Detection of endogenous levels of total PKM protein (including M1 and M2) also did not identify any significant difference between gp120 treated and untreated glioma cells (Supplemental Figure S4). However, colorimetric pyruvate kinase activity assays revealed a significant increase in pyruvate production in cells treated with gp120 for 7–10 days compared to untreated cells (Figure 5), indicating an increase in PKM2 enzymatic activity. This effect was not observed at a shorter time points of gp120 treatment (Supplemental Figure S1B). These findings indicate that the HIV glycoprotein gp120 increases the enzymatic activity of PKM2 in glioma cells without affecting total PKM2 protein levels.

Apoptosis inducers dexamethasone, temozolomide and puromycin were used to control the activity of glycolytic enzymes in glioma cells after apoptosis was induced (Supplemental Figure S5). Data demonstrate that after exposure to inducers of apoptosis, cells exhibited no significant increase in the activity of glycolytic enzymes, indicating that observed gp120-relatad activation of glycolytic enzymes is not associated with the apoptosis mechanism.

Despite the increase in protein expression for ENO2 and GAPDH, we did not observe a significant increase in the mRNA level of these enzymes. In the case of HXK, similar to the up regulation in protein levels, we observed an increase in HXK mRNA (Supplemental Figure S6), suggesting that gp120-induced expression of specific glycolytic enzymes in glioma cells can be regulated at both the transcriptional and translational levels.

**Figure 6:**
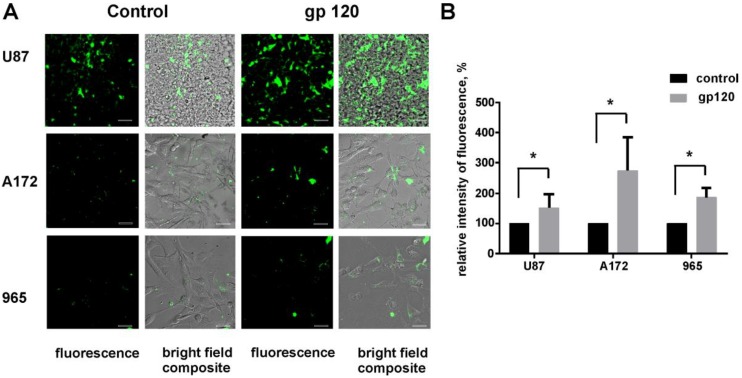
Gp120 increases glucose uptake in glioma cells. Glucose uptake assays were performed for untreated and gp120 treated for 7–10 days U87, A172 and 965 glioma cells. Confocal images showing the uptake of GluTracker reagent in the cytoplasm (**A**) and quantification of relative fluorescence intensity (**B**) are presented. Mean ± S.E. and significant differences between control and gp120 treatment (*) are shown (*p* < 0.05). Unpaired *t*-tests were used to determine the significance between groups. Five independent experiments (*n* = 5) were used for statistical analysis. Scale bar: 20 µm.

**Figure 7:**
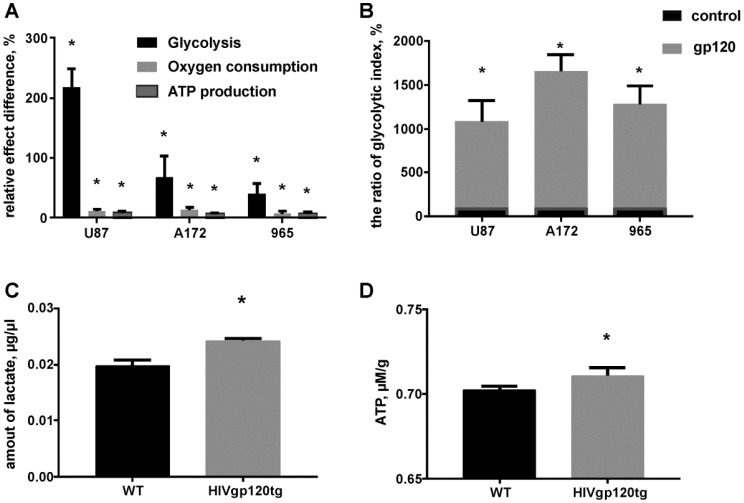
Gp120 causes a glycolytic shift in glioma cells. (**A**) Colorimetric/fluorometric assays were performed for multiplex detection of extracellular acidification, extracellular oxygen consumption and ATP production in U87, A172 and 965 glioma cells. (**B**) Quantification of intracellular rates of glycolytic index using extracellular acidification, oxygen consumption and glucose uptake measurements. Data presented as a percent deviation from the control. (**C**,**D**) Colorimetric assays were performed for detection of lactate (**C**) and ATP (**D**) in tumors generated in WT and HIVgp120tg mice by in-brain implantation of Gl261 cells. Mean ± S.E. and significant differences between control and gp120 treatment (*) are shown (*p* < 0.05). Unpaired *t*-tests were used to determine the significance between groups. Five independent experiments (*n* = 5) were used for statistical analysis.

To estimate the glycolytic activity in gp120 treated and untreated glioma cells we performed a combination of assays including glucose uptake assay, fluorometric oxygen consumption assays, glycolysis (extracellular acidification) assays and ATP production colorimetric assays. In all cell lines studies, we observed a significant increase in the glucose uptake ratio in gp120-treated versus untreated cells (Figure 6). Treatment with gp120 resulted in a 1.5 times increase in glucose uptake in U87 cells, 2.7 times increase in A172 cells and 2 times increase in 965 cells. Additionally, the study revealed a strong increase in glycolysis (extracellular acidification) of 50 to 200% in response to 7–10 days gp120 treatment in the three glioma cell lines tested, with the highest increase observed in U87 cells (200%) and the smallest response in 965 cells (50%) (Figure 7A). Similarly, gp120 treatment resulted in 6–10% increase in oxygen consumption and ATP production, indicating that oxidative phosphorylation is also affected by gp120 in glioma cells, although to a lower extent than the glycolytic pathway. By calculating the ratio of glycolytic indexes (lactate generation rate × glucose uptake rate)/oxygen consumption rate) we revealed a 10–15 times increase in treated glioma cells (Figure 7B), indicating a glycolytic shift in response to gp120.

Finally, we analyzed lactate and ATP production in glioma tumors generated in HIVgp120tg and WT mice. Similar to our results in glioma cell lines, we detected a significant increase in lactate production (21%) and a 2% increase in ATP production in tumor tissues extracted from the brains of HIVgp120tg animals (Figure 7C,D).

### 2.3. Inhibition of Glycolysis Eliminates the Stimulatory Effect of gp120 on Glioma Cell Growth

To investigate whether the increased proliferation we observed in glioma cells in response to gp120 was dependent on glycolysis we investigated the effect of sodium monofluorophosphate (FP), a competitive blocker of ENO2 and glycolysis, on glioma cell viability. Cytotoxic effect of FP on gp120-exposed and unexposed glioma cells was investigated in dose-response study with use of cell cycle analysis and trypan blue staining of dead cells (Supplemental Figure S7A,B). Treating glioma cells with a low (0.9 mm/mL) and a high (3.0 mg/mL) dose of FP resulted in an increased number of cells in G1 and a lower number of cells in the S phase. At 0.9 mg/mL FP the treatment resulted in a significant reduction of cells in the G2/M phase which correlates with a reduction in the S phase and the increase in G1. At the higher dose of 3 mg/mL, FP treatment increased the G2/M fraction indicating a cell cycle arrest. Based on these results, we selected the lower concentration of FP for further experiments as it showed no toxic effects while still capable of reducing PMK2 activity to control levels in cells treated with gp120 (Supplemental Figure S7C).

**Figure 8:**
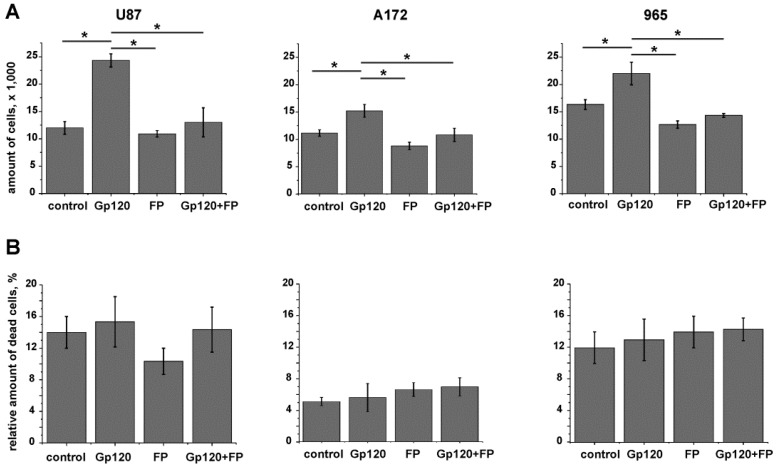
Sodium monofluorophosphate (FP) reverses the effect of gp120-induced proliferation in glioma cells. Experiments were performed in untreated glioma cells and cells continuously treated with gp120 for 10 days. U87 and A172 cell lines and 965 primary glioma cells were investigated. Cells were treated with FP, 0.9 mg/mL, for 24 h and live and dead cells were counted with the use of trypan blue staining. (**A**) Cell viability was evaluated as the total number of live cells grown in 24 h. (**B**) The proportion of dead cells was evaluated as the percentage of the total number of cells. Mean ± S.E. and significant differences from control (*) are shown (*p* < 0.05). Unpaired *t*-tests were used to determine the significance between groups. Six independent experiments (*n* = 6) were used for statistical analysis.

When glioma cells grown in medium supplemented with gp120 were treated with FP, the effect of increased viability observed in response to gp120 was reversed (Figure 8A). Because we did not observe significant cell death in response to FP treatment (Figure 8B), detected viability can be interpreted solely as a result of proliferation. This conclusion is confirmed by cell cycle analysis of gp120-treated and untreated glioma cells in the presence or absence of FP (0.9 mg/mL) (Supplemental Figure S7D). In gp120-exposed glioma cells treated with FP the cell cycle activity was partially reversed till the control level compare to gp120-unexposed cells treated with FP.

### 2.4. gp120 Causes Stimulation of Protein and Lipid Synthesis and a Reduction of Protein Degradation

In order to evaluate the metabolic pathways affected in glioma cells in response to gp120 we performed a metabolomic analysis. All metabolites differentially expressed in gp120 treated and untreated U87 cells are presented in Table 1.

These results revealed a significant reduction in the number of essential and nonessential amino acids, including valine, leucine, isoleucine, alanine and glycine in cells treated with gp120. Additionally, we observed a non-significant downregulation of tyrosine, cysteine and aspartate. Accordingly, canonical pathways identified by Ingenuity pathway analysis (IPA) predicted significant changes in aminoacyl–tRNA biosynthesis (*p* value = 8.56E-38). At the same time, a six-fold reduction in urea, the principal end product of protein catabolism, was observed. Thus, downregulation of these amino acids together with the reduction in urea levels predicts the activation of protein synthesis and reduction of protein degradation.

**Figure 9:**
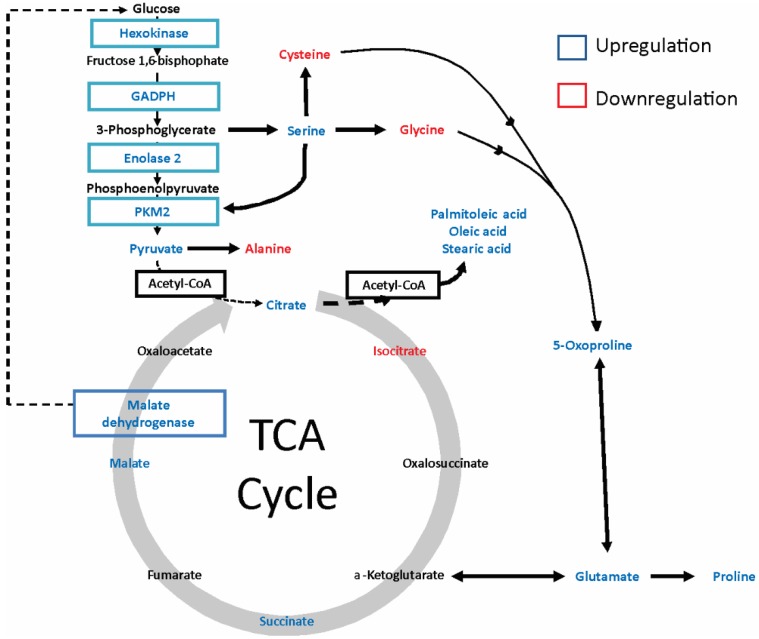
Schematic representation of the metabolic pathways altered in glioma cells by gp120 protein.

Additionally, we discovered an increase in serine (3.14 fold), glutamate (2.5 fold) and methionine (2.1 fold) in gp120-treated cells as well as a drastic increase in proline (23.7 fold) and tryptophan (15.6 fold). Since the biosynthesis of serine starts with 3-phosphoglycerate, an intermediate metabolite from glycolysis produced by GAPDH, which we previously observed to be upregulated in response to gp120, we conclude that gp120 stimulates the serine metabolic pathway through the stimulation of glycolysis, which was predicted to be significantly increased according to IPA canonical pathway analysis (*p* value = 6.04E-07) due to enhanced expression of ENO2, GAPDH and PKM2 (Figure 9). The elevation of lactate observed in gp120-treated cells also indicates an intensification of glycolysis and supports our conclusion.

Upregulation of 5-oxoproline together with a 3-fold reduction in glycine in gp120-treated cells was induced by activation of the γ-glutamyl cycle, which was predicted to be significantly shifted to the production of 5-oxoproline according to IPA canonical pathway analysis (*p* value = 1.44E-07). This finding is consistent with reports indicating that the deficit of glycine results in a metabolic switch in the γ-glutamyl cycle resulting in the production of 5-oxoproline but not glutathione [38,39]. Upregulation of glutamate together with proline indicates formation of a 5-oxoproline–glutamate–proline metabolic axis (Figure 9).

The IPA canonical pathway analysis also predicted a significant increase in TCA cycle turnover (*p* value = 4.2E-05) as revealed by the upregulation of its metabolic intermediates including citrate, succinate, and malate. Concomitant with these results, isocitrate was downregulated, suggesting an increase in fatty acid synthesis (see below). These results correlate with our data from oxygen consumption and ATP production assays and strongly support our conclusion that oxidative phosphorylation is increased in glioma cells in response to gp120.

U87 glioma cells treated with gp120 showed an upregulation of stearic, oleic, myristic and palmitoliec fatty acids. Considering that citrate is the primary substrate for the fatty acid synthesis pathway [40], the observed upregulation of citrate together with reduction of isocitrate and upregulation of fatty acids indicates the activation of fatty acid synthesis metabolism and the use of citrate for fatty acid synthesis in glioma cells in response to gp120.

The reduction of isocitrate was followed by upregulation of succinate and malate. The fact that glutamate was upregulated in gp120-treated cells, glutamate dehydrogenization and conversion to α-ketoglutarate with further entry into the TCA cycle is plausible and would explain the upregulation of downstream TCA cycle metabolites, such as succinate and malate.

To further confirm our data on metabolomics analysis and the predicted increase in protein synthesis in glioma cells in response to gp120 treatment we analyzed global protein synthesis by flow cytometry using an assay based on O-Propargyl-puromycin and further staining of truncated polypeptides with a fluorescent azide. Therefore, an increase in fluorescence detected by Flow Cytometry indicates an increase in global protein synthesis.

**Figure 10:**
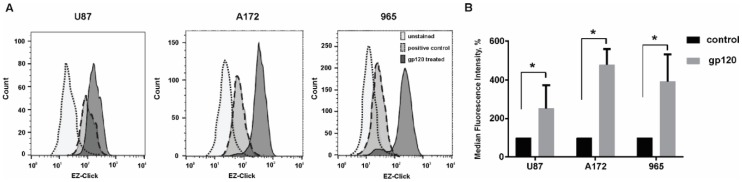
Gp120 increases global protein synthesis in glioma cells. EZClick Global Protein Synthesis Assay Kit (BioVision Inc, Milpitas, CA, USA), followed by flow cytometry analysis, was used for the identification of de novo synthesis of polypeptides. Experiments were performed for untreated glioma cells and cells continuously treated with gp120 for 7–10 days. U87 and A172 cell lines and 965 primary glioma cells were investigated. (**A**) Graphs represent the total distribution of stained cells in gp-120 treated and untreated glioma cell lines. (**B**) The relative median fluorescence intensity is calculated as a percentage of median fluorescence intensity in cells treated with gp120 relative to untreated cells. Mean ± S.E. and significant differences between gp120-treated and untreated groups (*) are shown (*p* < 0.05). Unpaired *t*-tests were used to determine the significance between groups. Four repeated experiments (*n* = 4) were used for statistical analysis.

Our results showed an increase in the fluorescence of U87, A172 and 965 glioma cell lines treated with gp120 for 7–10 days compared to untreated cells further confirming that in these cells, protein synthesis is increased in response to gp120 (Figure 10). Taken together with our data on metabolomics, amino acids composition and urea production, these results indicate that treatment with gp120 results in a strong activation of protein synthesis in glioma cells.

## 3. Discussion

Our study demonstrated that the HIV glycoprotein gp120 promotes proliferation, migration, survival and stimulates glycolysis in glioma cell lines. Increased glycolysis, also known as the Warburg effect, is characteristic of malignancy [41,42]. Upregulated glycolysis promotes unconstrained proliferation and invasion of tumor cells, providing the required glycolytic intermediary precursors for DNA, protein and lipid synthesis [43]. In Figure 9 we summarized the metabolic pathways that are altered in glioma cells in response to gp120. We observed an upregulation of the key glycolytic enzymes HXK, GAPDH and ENO2 in glioma cells treated with gp120. Despite the fact that we did not observe an upregulation of PKM2 protein levels—the enzyme that catalyzes the final step of glycolysis, converting phosphoenolpyruvate (PEP) to pyruvate—we found a significant increase in PKM2 activity and pyruvate synthesis, as well as an increase in the glycolytic index. These findings indicate that gp120 stimulates the glycolytic pathway in glioma cells.

HIV infects immune cells by binding to CD4, CCR5 and CXCR4 through its envelope protein gp120 [44,45,46,47]. Current evidence suggests that most, if not all, GBMs express CXCR4 and CCR5 [27,28,29] and these receptors are related to the survival, invasiveness, proliferation and resistance to the radio- and chemotherapy of glioma tumors [27,28,30,48,49,50]. It has been shown that activation of CCR5 and CXCR4 promotes a global shift towards anabolic metabolism and increased cell proliferation: increased glucose uptake, ATP production and enhanced glycolysis, associated with extracellular acidification [51,52,53]. Numerous viruses have been shown to cause significant alterations in the metabolism of the host cell, including HIV, hepatitis C, influenza, herpes simplex and human cytomegalovirus [54]. The production of the enveloped viruses requires additional fatty acid and nucleotide synthesis to progeny virions and these biosynthetic pathways are supported by the upregulation of glycolysis and the TCA cycle in a host cell [55,56,57]. It was noted that these changes in metabolic activity of infected cell are similar to oncogenic transformation [58,59], suggesting that the biosynthetic needs of a virally infected cell are similar to those of proliferating cells. Based on these reports, we assume that the gp120-induced activation of glycolysis and cell proliferation might result from activation of cell surface signaling molecules, such as CXCR4 or CCR5 and further-downstream signaling activation in glioma cells. It was reported that activation of CXCR4 and CCR5 leads to the activation of AKT [60,61,62] and promotes tumor proliferation [63]. Activated AKT phosphorylates multiple downstream targets including Glycogen Synthase Kinases (GSK3) and Mammalian Target of Rapamycin (mTOR) involved in glycogen metabolism, glucose homeostasis and protein synthesis regulation [64,65]. GSK3 is over activated in GBMs and the level of GSK3 phosphorylation is associated with increased tumor growth [66,67]. It is plausible that gp120 binding to CXCR4 and CCR5 activates AKT/GSK3 signaling resulting in upregulation of glycolysis in glioma cells. Reported downregulation of Phosphatase and Tensin Homolog (PTEN) in investigated glioma cell lines [68,69,70], upregulation of AKT signaling and high level of activity of GSK3, might provide the mechanistic ground for gp120-driven proliferation and metabolic switch specifically in gliomas but not in other investigated cancer cell lines. However, the detailed evaluation of underlying signaling mechanism is a subject for future studies.

PKM2, which we found to be activated in response to gp120 in glioma cells, is another critical player in the metabolic reprogramming of cancer cells. PKM2 can be translocated into the nucleus and induce cellular proliferation [71] and has been shown to be activated in cancerous tissues [72,73]. PKM2 is found as dimeric and tetrameric forms in which the tetramer has a high affinity and the dimer has a low affinity for phosphoenolpyruvate (PEP) [74]. Cancer cells preferentially express the less active form of PKM2 [75,76], leading to accumulation of glycolytic intermediates in upstream pathways and facilitating the formation of cell-building components. The dimer/tetramer ratio acts as a sensor that regulates metabolic synthesis and energy production from mitochondria [76]. Since our study revealed an increase in PKM2 activity in gp120-treated glioma cells without upregulation of the PKM2 protein, we propose that gp120 can affect the PKM2 dimer/tetramer ratio for the coordination of glycolysis with the cell cycle. This conclusion is supported by literature reports indicating that both fructose 1,6-bisphosphate and PEP, the product metabolites of HXK and ENO2, upregulated by gp120 in our study, promote reassociation of the dimer into a tetramer [77,78] and facilitate entry into the Krebs cycle and a decrease in lactate production. This is consistent with our results indicating activation of pyruvate kinase together with an insignificant increase in lactate.

Additionally, it has been shown that serine can bind to and activate PKM2 [79]. Our study established that there is an increase in serine in gp120-treated glioma cells (Table 1). Taken together with the increased in pyruvate kinase activity in these cells, we propose the existence of a serine activation loop in the glycolysis pathway (Figure 9). The biosynthesis of serine starts with the oxidation of 3-phosphoglycerate, the metabolite produced by GAPDH. Upregulation of GAPDH, which we observed in our study, provides 3-phosphoglycerate as a primary source for the serine biosynthesis pathway. In addition to directing activation of PKM2, serine is crucial for cancer growth and oncogenic transformation due to its participation in the biosynthesis of purines, pyrimidines, sphingolipids and several amino acids, including glycine and cysteine [80,81,82,83] and has been found to be upregulated in cancers [83].

Our results revealed a reduction in the amino acid levels in cells treated with gp120. Together with the reduction in urea (Table 1) and the upregulation of molecules involved in protein synthesis, previously identified by our group [32], such as the RPS2, RPS13, RPS15 ribosomal proteins and number of initiation and elongation factors as eIF4A1, eIF4G1, PABPC1 and eEFG1, these findings indicate the predominance of anabolic processes over catabolic processes and active protein synthesis. It has been shown that glutamine uptake is essential for growth in a number of cancers [84,85,86,87,88,89]. Glutamine, through glutamate, is a source of α-ketoglutarate in the TCA cycle, glutathione in redox homeostasis and citrate by reductive carboxylation to form lipids and glucosamines [90]. Tumor cells tend to have a large pool of glutamate and this pool is maintained by the cell’s ability to convert glutamine into glutamate through glutaminases [91]. The upregulated glutamate observed in gp120-treated glioma cells indicates the increased use of TCA cycle metabolites for synthesis of lipids and amino acids and replenishment of the TCA cycle through the glutamate–ɑ-ketoglutarate pathway. An increase in glutamate in gp120-treated cells can result from the increased uptake of glutamine and further conversion into glutamate, such as through the synthesis from 5-oxoproline, which is also upregulated in gp120-treated cells. Recent studies have shown that 5-oxoproline participates in the regulation of Na^+^-dependent transport of glutamate [92,93] and may contribute to the increased glutamate uptake by gp120-treated glioma cells.

We detected a 24-fold increase in proline in gp120-treated cells. Proline can be synthesized from glutamine [94] and the metabolism of proline serves as a source of energy during stress, provides signaling reactive oxygen species for epigenetic reprogramming and regulates redox homeostasis [86]. The critical role of proline biosynthesis in maintaining pyridine nucleotide levels by connecting the proline cycle to glycolysis and to the oxidative arm of the pentose phosphate pathway have also been shown [95]. Proline biosynthesis activity has been associated with tumor cell growth, resistance to oxidative stress and energy production [96,97]. Based on this we can assume, that the increase in proline synthesis in gp120-treated glioma cells might be associated to increased proliferation and survival through reprogramming of the glutamine and pyridine pathways.

Abnormal cellular lipid metabolism also plays an important role in cancer. Fatty acid biosynthesis is restricted to a subset of tissues, including liver, adipose and lactating breast tissues. However, reactivation of lipid biosynthesis has been reported in cancers [98,99] and is associated with cancer growth and invasiveness [100,101]. Fatty acids are the major building blocks for the synthesis of phosphoglycerides and are structural components of biological membranes, contributing to cancer cell proliferation. In our study, we detected the upregulation of stearic, oleic, myristic and palmitoleic fatty acids in glioma cells in response to gp120 which together with our other findings supports the increased proliferation and survival in gp120 treated glioma cells.

Another important finding from this study is related to the substantial 15-fold increase in tryptophan in gp120-treated cells. Many cancers drive tryptophan consumption [102] and it has been shown that the primary product of tryptophan metabolism, kynurenine, is an endogenous ligand for the aryl hydrocarbon receptor, which mediates invasive tumor growth and the evasion of immunity [102,103,104]. The autocrine binding of kynurenine to the aryl hydrocarbon receptor in cancer cells causes the transcriptional activation of genes related to tumor invasiveness [105]. In patients with cancer, the upregulation of indoleamine 2,3-dioxygenase, an enzyme that generate kynurenine from tryptophan, is associated with a poor prognosis [106]. The increased consumption of tryptophan observed in gp120-treated glioma cells may be related to the increased migration of these cells.

## 4. Materials and Methods

### 4.1. Cell Culture

U87 and A172 human glioma cell lines were obtained from the American Type Culture Collection (Manassas, VA, USA). The GL261 glioma cell line derived from C57BL/6 mice was obtained from the NCI (Frederick, MD, USA). The 965 primary human glioma cell line was obtained from a resected GBM tumor mass in the laboratory of Dr. Quinones-Hinojosa, Mayo Clinic. Clinical data for 965 primary cell lines have been described and analyzed in detail previously [68,107,108]. Cells were cultured in Dulbecco’s modified Eagle medium (DMEM) supplemented with 10% fetal calf serum and 50 U/mL penicillin/50 µG/mL streptomycin and maintained in a humidified atmosphere of 5% CO2/95% air at 37 °C. Cultures used in this study underwent less than sixteen passages.

### 4.2. Animals

All procedures involving rodents were conducted in accordance with the National Institutes of Health regulations concerning the use and care of experimental animals. All procedures involving animals were approved by Universidad Central del Caribe Institutional Animal Care and Use Committee (protocol #036-214-14-01-PHA from 4 August 2016). All efforts were made to minimize suffering.

HIVgp120tg mice were kindly provided by Dr. Marcus Kaul (Sanford Burnham Prebys Medical Discovery Institute, San Francisco, CA, USA) [35,36]. C57Bl/6 mice were purchased from the Jackson Laboratory. C57Bl/6 and HIV gp120tg mice were crossbred and animals heterozygous for HIV gp120 and wild type littermates were used as experimental and control groups correspondingly. Genotyping was performed according to the previously published protocols [36].

### 4.3. Intracranial Implantation of Glioma Cells

All surgeries were performed under isoflurane anesthesia and all efforts were made to minimize suffering. GL261 glioma cells were implanted into the right cerebral hemisphere of 12–16 week old C57BL/6 mice. Implantation was performed according to the protocol that we described earlier [109]. Briefly, mice were anesthetized with isoflurane and a midline incision was made on the scalp. At stereotaxic coordinates of bregma, 2 mm lateral, 1 mm caudal and 3 mm ventral a small burr hole (0.5 mm diameter) was drilled on the skull. 1 μL of cell suspension (2 × 104 cells/μL in PBS) was delivered at a depth of 3 mm over 2 min. Sixteen days following injection, animals were anesthetized with pentobarbital (50 mg/kg) and transcardially perfused with PBS followed by 4% paraformaldehyde (PFA). Brains were removed and postfixed in 4% PFA/PBS for 24 h at 4 °C, followed by 0.15 M, 0.5 M and 0.8 M sucrose at 4 °C until fully dehydrated. Brains were then frozen-embedded in Cryo-M-Bed embedding compound (Bright Instrument, Huntingdon, England) and cut using a Vibratome UltraPro 5000 cryostat (American Instrument, Haverhill, MA, USA). 

### 4.4. Tumor Size Evaluation

15 μm coronal frozen sections encompassing the entire tumor were stained with Hematoxylin & Eosin. Tumor size was calculated as sum of tumor area × section thickness for each section containing a tumor.

### 4.5. Survival Analysis

GL261 glioma cells were implanted into HIVgp120tg and WT littermate mice. Animals were inspected daily and body weight loss of 15%, decreased activity/responsiveness, abnormal posture or any neurological disorders signs are to be a subject for euthanasia. Time between tumor bearing and animal death was recorded.

### 4.6. In Vitro Viability Assay

Glioma cells were plated in petri dishes at 200,000 cells per dish and incubated for 10 days with and without gp120 (100 ng/mL). The cells were then harvested, stained with trypan blue and the total number of live and dead cells determined by cell counting.

### 4.7. Migration Assay

Migration assays were performed using Fluoroblok inserts (8-µM pore size, VWR Scientific). Serum-starved cells (30,000) were placed on the insert membrane and the assays were performed following the addition of medium containing 5% serum to the lower compartment. After 5 h, the cells were fixed with methanol and stained with propidium iodide. The number of cells that had migrated to the lower compartment was determined by counting the number of fluorescent cells.

### 4.8. Cell Cycle Assays

Cells were harvested, fixed in 70% ethanol, re-suspended in PBS containing 1 µg/ml 7-AAD (Bio-Rad Laboratories, Hercules, CA, USA) and 0.2 mg/mL RNase A (Sigma-Aldrich, St. Louis, MO, USA), incubated for 30 min at 37 °C in the dark and analyzed with a FACSCanto II flow cytometer (Becton Dickinson, San Jose, CA, USA). The percentage of cells in G0/G1, S and G2/M phases was determined from the DNA content using FlowJo data analysis software v.10 (Ashland, OR, USA).

### 4.9. Western Blot Analysis

Clarified cell lysates separated on 10% SDS-PAGE gels were transferred to PVDF membranes and probed with mouse anti-ENO2 antibody (Santa Cruz Biotechnology, Dallas, TX, USA; #SC-21738) and rabbit polyclonal anti-PKM2, anti-GAPDH and anti-HXK antibodies (Cell Signaling, Danvers, MA, USA; #4053, #5174 and #2024, respectively), diluted 1:1000, followed by the secondary antibodies (Sigma-Aldrich, Saint Louis, MO, USA; #A9169). Detection was performed with enhanced chemiluminescence methodology (SuperSignal^®^ West Dura Extended Duration Substrate; Pierce, Rockford, IL, USA) and the intensity of the signal was measured using a gel documentation system (Versa Doc Model 1000, Bio Rad). The intensity of the chemiluminescent signal was corrected for minor changes in protein content after densitometry analysis of the India ink-stained membrane.

### 4.10. Pyruvate Kinase, Hexokinase and Glyceraldehyde 3-Phosphate Dehydrogenase Activity Assays

Intracellular pyruvate, HXK and GAPDH activity was measured in cell lysates with the Pyruvate Kinase Activity Colorimetric/Fluorometric Assay kit (BioVision, Milpitas, CA, #K709-100), HXK Activity Fluorometric Assay Kit (Abcam, Cambridge, MA, USA, #ab211103) and GAPDH Activity Colorimetric Assay Kit (Abcam, Cambridge, MA, USA, #ab204732) according to the manufacturer’s protocol. In each experiment, 5,000 cells were used and their OD at 570 nm (for Pyruvate Kinase Assays), 450 nm (for GAPDH assays), or fluorescence at Ex/Em = 535/587 nm measured with a Perkin Elmer Wallac 1420 Victor2 Microplate Reader. Standard curves were used to determine the concentration of pyruvate, HXK, or GAPDH in the sample from the numeric colorimetric/fluorometric data. Enzyme’s activity was calculated as the amount of product produced in the sample in 10 min.

### 4.11. Glucose Uptake Assays

Glucose uptake was measured with the cell-based Glucose Uptake Assay Kit (Abcam, Cambridge, MA, USA, #ab204702) according to the manufacturer’s protocol. 50,000 cells were seeded on coverslips one day before starting the assay. Cells were incubated with glucose uptake mix containing fluorescent GluTracker reagent for 30 min and immediately visualized using an Olympus Fluoview FV1000 confocal microscope (Olympus, Japan) with 40× oil immersion objective and FITC excitation—emission filter set (absorption maximum at 494 nm and emission maximum of 521 nm). The fluorescent images were processed using ImageJ software.

### 4.12. Glycolysis and Extracellular Oxygen Consumption Assays

Comparative measurements were taken with Glycolysis Assay (Abcam, Cambridge, MA, USA, #ab197244) and Extracellular Oxygen Consumption Assay (Abcam, Cambridge, MA, USA, #ab197243) according to the manufacturer’s protocols. Cells were seeded in a 96-well plate at a density of 5000 cells/well. 24 h later the cell culturing medium was replaced with Glycolysis Assay Reagent or Extracellular Oxygen Consumption Reagent and the assay’s signals were measured simultaneously with a Perkin Elmer Wallac 1420 Victor2 Microplate Reader using Ex/Em = 380/615 nm.

### 4.13. ATP and Lactate Assays

ATP and L-Lactate were measured in cell lysates with the ATP Assay Kit (Abcam, Cambridge, MA, USA, #83355) and L-lactate Assay Kit (Abcam, Cambridge, MA, USA, #65331) according to the manufacturer’s protocol. Cultured cells or tissue lysates were used and their OD at 570 nm measured with a Perkin Elmer Wallac 1420 Victor2 Microplate Reader. A standard curve was used to determine the concentration of ATP and lactate in the sample from the numeric colorimetric data.

### 4.14. Metabolomics Analysis

Metabolites were extracted using an optimized protocol [110]. Cultured cells were scraped in MeOH/H2O (85:15), sonicated and centrifuged at 13,000× g. The supernatant was dried in a SpeedVac (Savant AS160, Farmingdale, NY, USA), followed by methoxyamination in a 20-mg/mL methoxyamine hydrochloride in pyridine (Sigma-Aldrich, St. Louis, MO, USA) and trimethylsilylation in N-methyl-N-trimethylsilyl-trifluoroacetamide (MSTFA + 1% trimethylchlorosilane (TMCS), ThermoFisher Scientific, Waltham, MA, USA). The supernatants were dissolved 3/50 in hexane and applied to a GC-2010 gas chromatograph (Shimadzu Scientific, Columbia, MD, USA) with an AOC-20i auto-injector in split mode (split ratio, 15). The analytes were fractionated on a fused-silica capillary RXI-5MS column (0.25 mm inner diameter, 0.25 μm D.F.; 30 m; Restek Bellefonte, PA, USA). The oven temperature was set to increase from 100 °C to 290 °C at 8 °C/min. Mass spectra were obtained on a Shimadzu GCMS-QP2010 mass spectrometer (EI, 70 eV, ion source temperature, 200 °C) in scan mode between 35 and 700 amu. The data obtained were processed using GCMS Solution Post-run Analysis software (Shimadzu Corp) for metabolite identification by comparison with the NIST08 spectral mass library (National Institute of Standards and Technology, Gaithersburg, MD, USA) using the NIST MS Search Program 2.0, mass spectral databases and AMDIS Version 2.71 deconvolution software (Automated Mass Spectral Deconvolution and Identification System, www.amdis.net) [111]. Peaks were integrated (maximum peak number, 200; width time, 2 s; smoothing method, standard) and manually checked. The peak intensities were quantified as the fold-change relative to control and the statistical significances were analyzed using Student’s *t*-test. *p*-values < 0.05 were considered significant. Metabolomics analysis was conducted using the IPA core analysis of metabolites (QIAGEN, Redwood City, CA, USA). Data sets containing metabolite identifiers (KEGG IDs) and metabolic enzyme protein identifiers (UNIPROT IDs), including their corresponding fold-change values relative to control, were mapped together with their corresponding objects in the Ingenuity Knowledge. Fisher’s exact test was used to calculate the probability that each ID set was enriched. Only the biological functions/pathways (Bonferroni’s corrected *p* value < 0.05) were considered significantly enriched.

### 4.15. Protein Synthesis Assay

EZClick Global Protein Synthesis Assay Kit (BioVision Inc., Milpitas, CA, USA, #K715-100) was used according to the manufacturer’s protocol. Briefly, 500,000 cells were seeded in 60mm petri dishes 24 h prior to the assay followed by addition of the Protein Label for 1 h. Cells were harvested, fixed and stained with Fluorescent Azide followed by Flow Cytometry analysis, using a BD-FACSCanto II flow cytometer (Becton Dickinson, San Jose, CA, USA). Flow cytometry data was analyzed using FlowJo data analysis software v.10 (Ashland, OR, USA).

### 4.16. Chemicals and Reagents

Sodium monofluorophosphate was obtained from Santa Cruz Biotechnology, Inc., Dallas, TX, USA, cat. #SC-264320

### 4.17. Statistical Analysis

Results are expressed as mean ± standard deviation (SD). The statistical probability was calculated using GraphPad software. Unpaired t-tests or one-way ANOVA tests followed by Tukey’s post-hoc test were used to determine significance between groups. *p*-values < 0.05 were considered as significant.

## 5. Conclusions

The HIV envelope protein gp120 stimulates glioma cell growth and glycolytic pathways through upregulation of key glycolytic enzymes, increased pyruvate kinase activity and glucose up-take. In conjunction with increased glycolysis, gp120 stimulates protein and fatty acid synthesis in glioma cells. As a subject for future studies, the signaling pathways initiated by gp120-activated cell surface receptors that may lead to the activation of glycolytic pathways in gliomas should be further investigated as a potential strategy to identify putative drug targets for glioma tumors in HIV infected patients. Additionally, the effect of whole HIV on glioma cell metabolism should be analyzed together with the effect of purified gp120 in vitro and in vivo. 

## Figures and Tables

**Table 1:** Metabolite compounds that were deregulated in gp120-treated U87 glioma cells compared to untreated cells according to GCMS/MS metabolomics analysis. * Cells were treated with gp120 for 10 days. Compounds highlighted in bold have values that are significantly different from the untreated group (*p* < 0.05).

ID HMDB	Compound ID	Retention Time (min)	Fold Change	*p* Value (*t* Test)	Characteristic Fragment Ions	Up/Down Regulation
MDB00190	L-lactic acid	10.27	1.612	2.50E-01	73,147,261	+
**HMDB00161**	**L-alanine**	**10.99**	**0.223**	**1.90E-02**	**73,158,232**	**−**
**HMDB00123**	**glycine**	**11.30**	**0.281**	**3.37E-02**	**73,147,218**	**−**
**HMDB00294**	**UREA**	**12.60**	**0.165**	**9.25E-05**	**104,147,231**	**−**
**HMDB00883**	**L-valine**	**12.72**	**0.357**	**4.53E-02**	**73,186,260**	**−**
**HMDB00687**	**L-leucine**	**13.29**	**0.236**	**1.32E-02**	**73,200,274**	**−**
**HMDB00172**	**L-isoleucine**	**13.74**	**0.013**	**1.29E-02**	**73,200,274**	**−**
**HMDB00162**	**L-proline**	**14.25**	**23.786**	**2.70E-02**	**73,184,258**	**+**
**HMDB00267**	**5-oxoproline**	**16.61**	**2.243**	**1.41E-02**	**73,272,300**	**+**
**HMDB00696**	**L-methionine**	**16.83**	**2.094**	**2.42E-02**	**73,218,292**	**+**
**HMDB00187**	**L-serine**	**17.11**	**3.14**	**2.59E-03**	**73,288,390**	**+**
HMDB00812	N-acetyl-L-aspartic acid	17.30	0.728	7.33E-01	73,346	−
HMDB00167	L-Threonine	17.51	0.993	6.35E-01	73,303,404	−
HMDB00806	Myristic acid	18.18	4.479	2.25E-01	75,285	+
HMDB00159	L-phenylalanine	18.32	0.899	9.85E-01	73,234,302	−
**HMDB00254**	**Succinic acid**	**14.1**	**1.723**	**2.92E-01**	**73,147,289**	**+**
HMDB00156	Malic acid	18.53	1.73	0.28091	73,115,419	+
HMDB00191	L-aspartic acid	19.03	0.783	2.63E-01	73,302,418	−
HMDB00574	L-cysteine	19.62	0.633	1.84E-01	73,378,406	−
**HMDB03229**	**Palmitoleic acid**	**20.16**	**3.205**	**1.85E-02**	**75,129,311**	**+**
**HMDB00123**	**L-glutamic acid**	**20.33**	**2.501**	**4.10E-02**	**73,272,432**	**+**
HMDB00220	Palmitic acid	20.43	1.093	8.04E-01	75,129,313	+
HMDB00641	L-glutamine	21.91	1.05	6.64E-01	73,147,431	+
**HMDB00207**	**Oleic acid**	**22.27**	**4.44**	**4.41E-02**	**75,129,339**	**+**
**HMDB00827**	**Stearic acid**	**22.50**	**3.63**	**3.28E-03**	**75,129,341**	**+**
HMDB00094	Citric acid	23.84	1.506	5.22E-01	73,459,591	+
HMDB00158	L-tyrosine	24.05	0.846	9.93E-01	73,302	−
**HMDB00929**	**L-tryptophan**	**24.49**	**15.672**	**1.81E-03**	**73,302,375**	**+**
HMDB01874	Isocitric acid	24.60	0.312	7.72E-01	73,345	−
HMDB00067	Cholesterol	37.20	1.010	4.59E-01	75,443	+

## Supplementary Materials

The following are available online at http://www.mdpi.com/2072-6694/10/9/301/s1, Figure S1. Continuous treatment with gp120 increases cell viability and activity of Pyruvate Kinase (PKM2) in U87 cells in 7–10 days; Figure S2. Viability assays were performed for glioma U87 (ATCC, #HTB-14), prostate PC-3 (ATCC, #CRL-1435), ovarian UACC-2727 (ATCC, #CRL-3192), Lewis lung LLC1 (ATCC, #CRL-1642), and Jurkat T cells (ATCC, #CRL-2899) tumor cell lines; Figure S3. Immunofluorescent detection of gp120 (green) and Glial Fibrillary Acid Protein (GFAP, red) was performed on frozen HIVgp120tg mouse brain sections containing the tumor area; Figure S4. Western blot evaluation of expression levels of Pyruvate Kinase M1/2 (PKM1/2) in U87 glioma cells; Figure S5. Induction of apoptosis is not associated with the activation of glycolytic enzymes in glioma cells; Figure S6. The relative expression of Hexokinase (HXK), Enolase 2 (Eno2) and Glyceraldehyde-3-Phosphate Dehydrogenase (GAPDH) mRNA was detected by qRT-PCR in U87 and A172 glioma cells; Figure S7. Sodium monofluorophosphate (FP) eliminates the stimulatory effect of gp120 in glioma cell proliferation and Pyruvate Kinase (PKM2) activity.
