# Supplementary material for: HIV-1 Envelope Protein gp120 Promotes Proliferation and the Activation of Glycolysis in Glioma Cell
Source: Cancers (Basel). 2018 Sep 1;10(9):301. doi: 10.3390/cancers10090301 (PMC6162763; doi:10.3390/cancers10090301)
Supplement: Supplementary file 1 [file cancers-10-00301-s001.pdf]

Supplementary Materials: HIV-1 Envelope Protein gp120 Promotes Proliferation and the Activation of Glycolysis in Glioma Cell

Gabriel Valentín-Guillama, Sheila López, Yuriy V. Kucheryavykh, Nataliya E. Chorna, Jose Pérez, Jescelica Ortiz-Rivera, Michael Inyushin, Vladimir Makarov, Aníbal Valentín-Acevedo, Alfredo Quinones-Hinojosa, Nawal Boukli, Lilia Y. Kucheryavykh

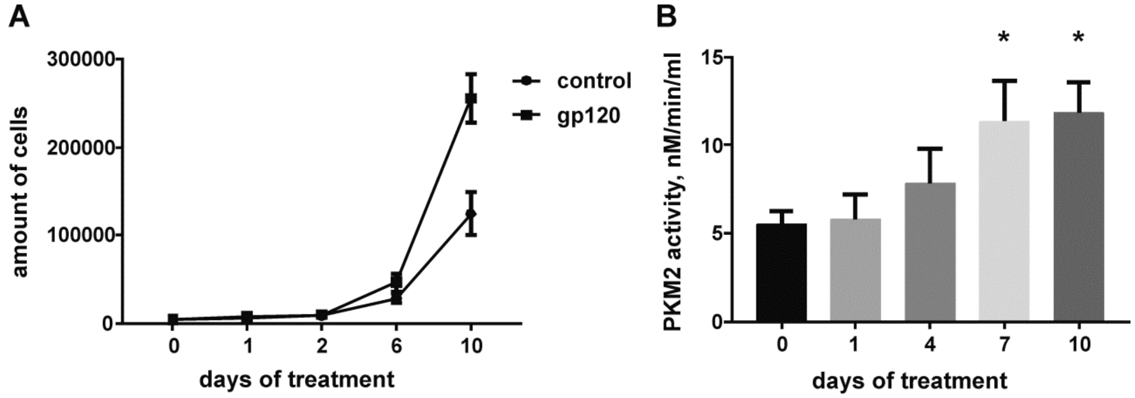

Figure S1. Continuous treatment with gp120 increases cell viability and activity of Pyruvate Kinase (PKM2) in U87 cells in 7–10 days. Viability assays based on trypan blue staining and direct cell count of live cells (**A**) and colorimetric PKM2 activity assays (**B**) were performed for untreated U87 cells and cells treated with gp120 for 0, 1, 2, 6, and 10 days. PKM2 activity was calculated as an amount of pyruvate produced in a sample in 10 min. Mean ± S.E. and significant differences between gp120-treated and untreated groups (*) are shown ($p < 0.05$). Unpaired *t*-tests were used to determine the significance between groups. 4 independent experiments ($n = 4$) were performed.

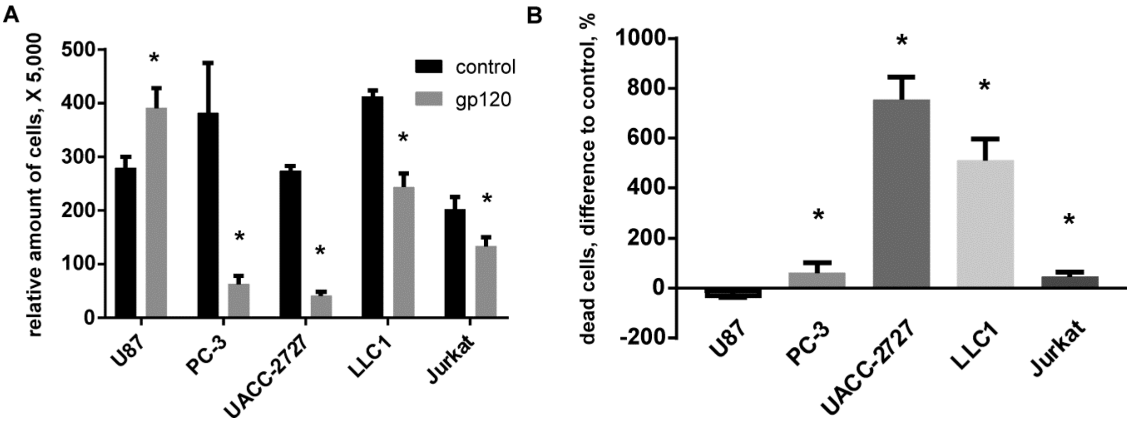

Figure S2. Viability assays were performed for glioma U87 (ATCC, #HTB-14), prostate PC-3 (ATCC, #CRL-1435), ovarian UACC-2727 (ATCC, #CRL-3192), Lewis lung LLC1 (ATCC, #CRL-1642), and Jurkat T cells (ATCC, #CRL-2899) tumor cell lines. Untreated cancer cells and cells continuously treated with gp120 for 48hours were used, except for Jurkat cells, which were treated for 24 h. Live and dead cells were counted with the use of trypan blue staining. (**A**) Cell viability was evaluated as the total number of live cells. (**B**) Amount of dead cells presented as deviation from the control after gp120 treatment. Mean ± S.E. and significant differences from control (*) are shown ($p < 0.05$). Unpaired *t*-tests were used to determine the significance between groups. Four independent experiments ($n = 4$) were used for statistical analysis.

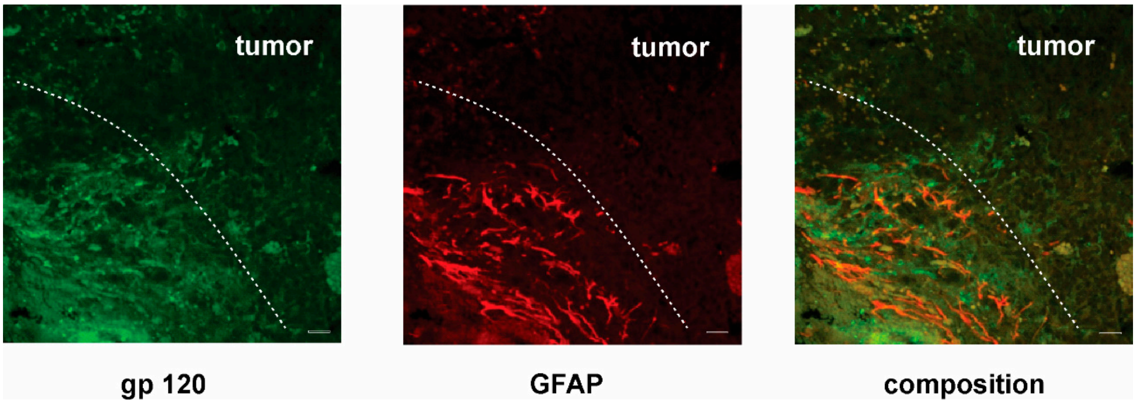

Figure S3. Immunofluorescent detection of gp120 (green) and Glial Fibrillary Acid Protein (GFAP, red) was performed on frozen HIVgp120tg mouse brain sections containing the tumor area. GL261 glioma cells were implanted into the brains of HIVgp120tg mice and grown for 16 days. Photographs show the expression of gp120 in the tumor and surrounding healthy tissue. The dash line outlines the border of tumor. The frozen sections were blocked with 5% normal goat serum/5% normal horse serum (Vector lab) and incubated with the primary antibody of interest overnight at 4 °C, followed by corresponding fluorescently-tagged secondary antibodies (Vector Lab) and visualized using an Olympus Fluoview FV1000 confocal microscope. Scale bar: 20 µm.

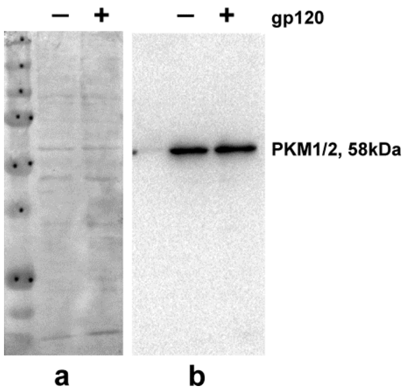

Figure S4. Western blot evaluation of expression levels of Pyruvate Kinase M1/2 (PKM1/2) in U87 glioma cells. (**A**) India Ink staining was used as a loading control for densitometry analysis. (**B**) Rabbit antibody against PKM1/2 (Cell Signaling, Danvers, MA, USA, #3190) detects endogenous levels of total PKM (including M1 and M2) protein, dilution 1:500, followed by anti-rabbit-conjugated immunoglobulins (Sigma-Aldrich Co, St. Louis, MO, USA) were used. Predicted band size: 58 kDa.

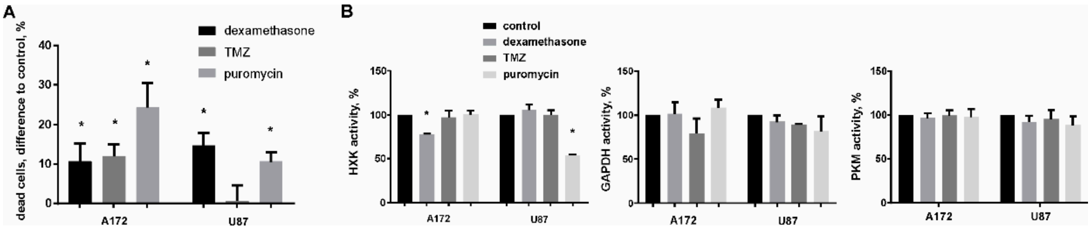

Figure S5. Induction of apoptosis is not associated with the activation of glycolytic enzymes in glioma cells. Dexamethasone (4 µg/ml, Sigma Aldrich, St. Louis, MO, USA, #D4902), temozolomide (TMZ, 150 µM, Sigma Aldrich, St. Louis, MO, USA, #T2577), and puromycin (2 µg/ml, Sigma Aldrich, St. Louis, MO, USA, #P8833) were used as apoptosis inducers. A172 and U87 cells were treated with dexamethasone or puromycin for 24 h, while TMZ was applied for 48 h before the study. Selected concentrations of the inducers of apoptosis resulted in no more than 20% of cell death compared to the control at the moment of the assays. (**A**). Viability analysis with use of trypan blue staining was performed for glioma cells. The graph represents the percent difference of dead cells

compared to control. (**B**) Colorimetric/fluorometric hexokinase (HXK), glyceraldehyde 3-phosphate dehydrogenase (GAPDH), and pyruvate kinase M (PKM) activity assays were performed in untreated glioma cells and cells treated with apoptosis inducers. Kinase activity was calculated as the amount of product (NADPH, NADH, and pyruvate, respectively) produced in a sample in 10 min. Mean ± S.E. and significant differences between control and treatment (*) are shown ($p < 0.05$). Unpaired *t*-tests were used to determine the significance between groups. Three independent experiments ($n = 3$) were used for statistical analysis.

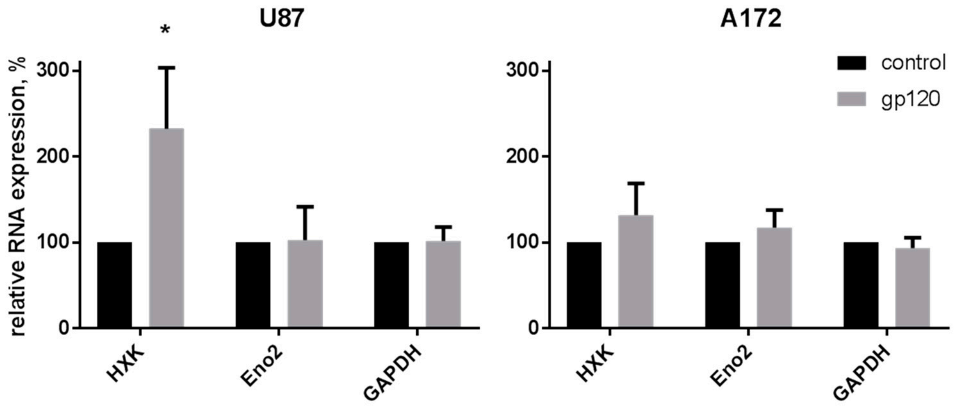

Figure S6. The relative expression of Hexokinase (HXK), Enolase 2 (Eno2) and Glyceraldehyde-3-Phosphate Dehydrogenase (GAPDH) mRNA was detected by qRT-PCR in U87 and A172 glioma cells. The study was performed for glioma cells treated with gp120 for 7 days. Mean ± S.E. and significant differences between gp120-treated and untreated cells (*) are shown ($p < 0.05$). Unpaired *t*-tests were used to determine the significance. $n = 3$.

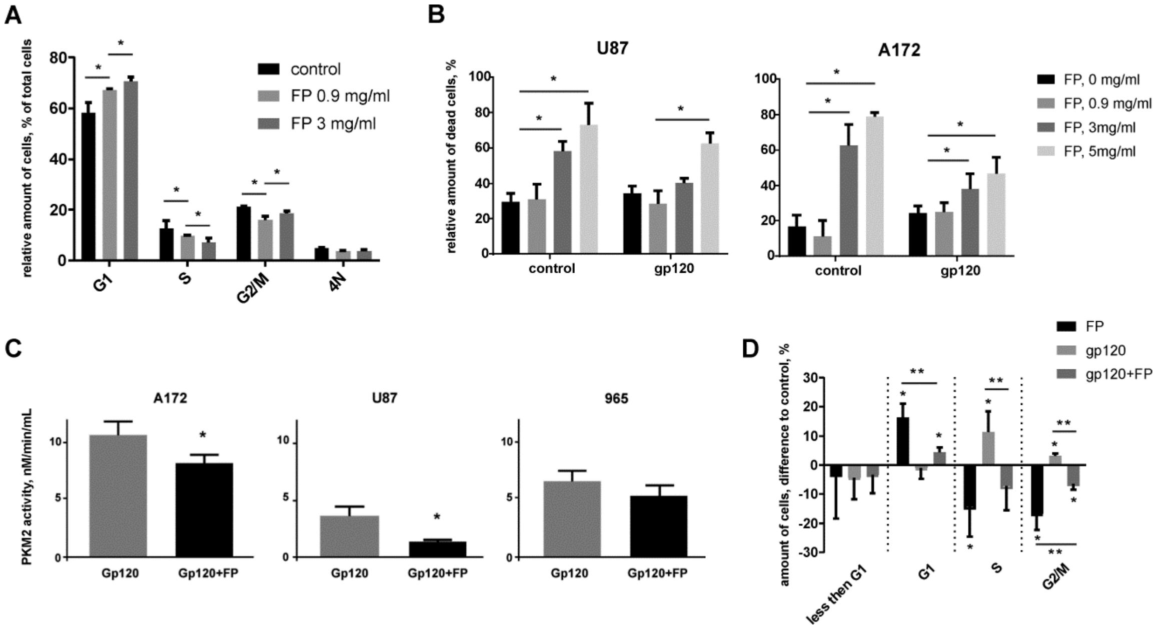

Figure S7. Sodium monofluorophosphate (FP) eliminates ~~eliminates~~ the stimulatory effect of gp120 in glioma cell proliferation and Pyruvate Kinase (PKM2) activity. (**A**) Cell cycle of A172 glioma cells exposed to different concentrations of FP for 24 h and stained with 7-aminoactinomycin D (7AAD) was analyzed by flow cytometry. The percentage of cells in the G0/G1, S and G2/M phases was determined based on DNA content. (**B**) Viability assays with use of trypan blue staining were performed for glioma cells treated with gp120 for 7 days and ~~and~~ untreated cells. FP was applied ~~for~~ 24 h prior to the performing the assays. Relative amount of dead cells compared to the total amount of cells is presented. (**C**) Colorimetric pyruvate kinase activity assays were performed for glioma cells treated with gp120 for 10 days. FP (0.9 mg/ml) was applied for 24 h before the assays. (**D**) Flow

cytometry analysis was performed for untreated glioma cells and cells continuously treated with gp120 for 7 days, as described in (**A**). FP (0.9 mg/ml) was applied for 24 h before the analysis. The graph represents the percent difference of cells in the G0/G1, S, and G2/M phases compared to control. Mean ± S.E. and significant differences between groups (*) are shown ($p < 0.05$). Unpaired *t*-tests were used to determine the significance. $n = 3$.

© 2018 by the authors. Licensee MDPI, Basel, Switzerland. This article is an open access article distributed under the terms and conditions of the Creative Commons Attribution (CC BY) license (http://creativecommons.org/licenses/by/4.0/).
